# Supplementary material for: Improvement in model flexibility reveals signaling pathway in T cell responses to pulsatile stimuli
Source: NPJ Syst Biol Appl. 2026 Jun 2;12:129. doi: 10.1038/s41540-026-00741-z (PMC13526819; doi:10.1038/s41540-026-00741-z)
Supplement: Supplementary file 1 [file 41540_2026_741_MOESM1_ESM.pdf]

1 Supplemental material

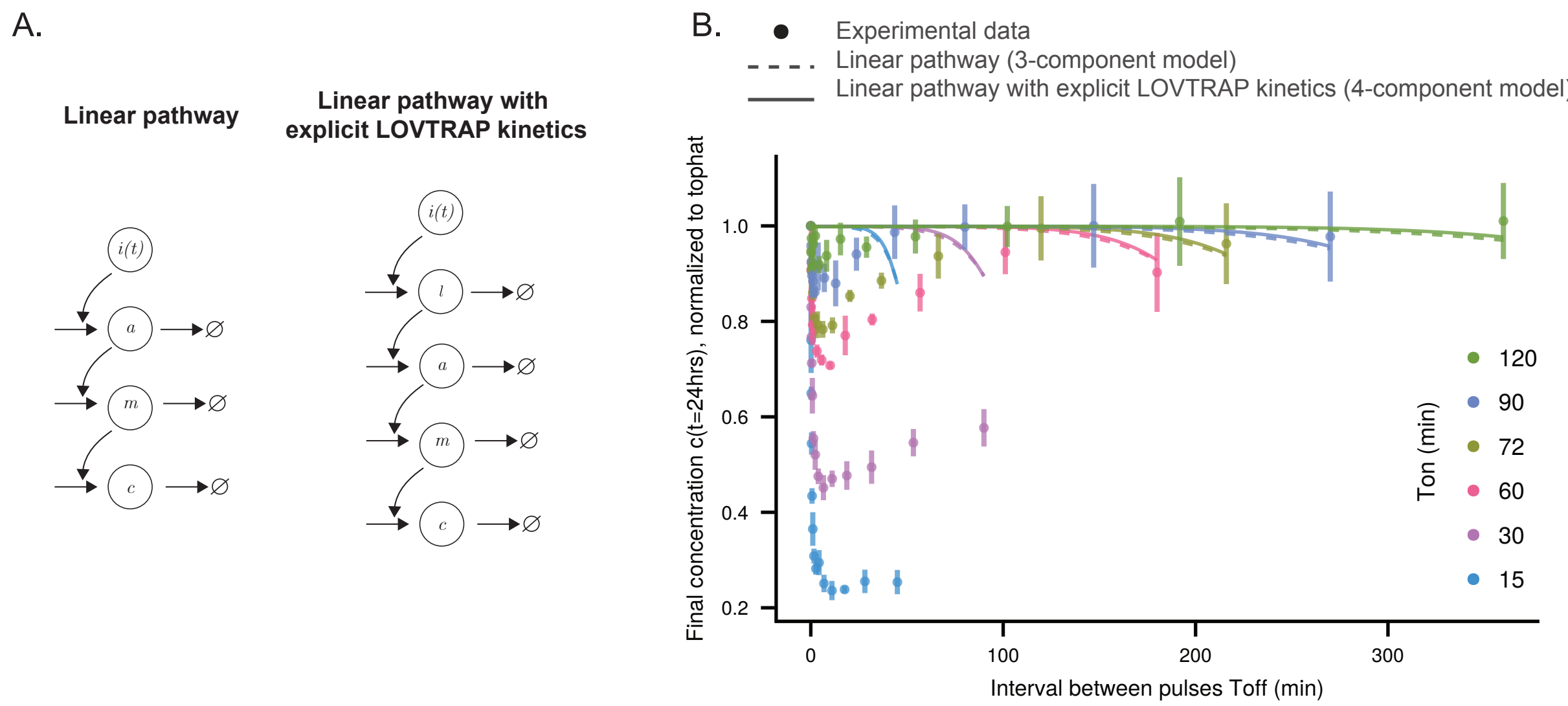

Figure S1: Linear pathway models, with or without explicit LOVTRAP dynamics, fail to explain the frequency response data. A Pathway schematics for linear pathway and linear pathway with explicit LOVTRAP kinetics. B Best-fit frequency response of linear pathway models. Solid: Linear pathway with explicit LOVTRAP dynamics (4-component model); dashed: linear pathway (3-component model). Plot symbols show data from [10].

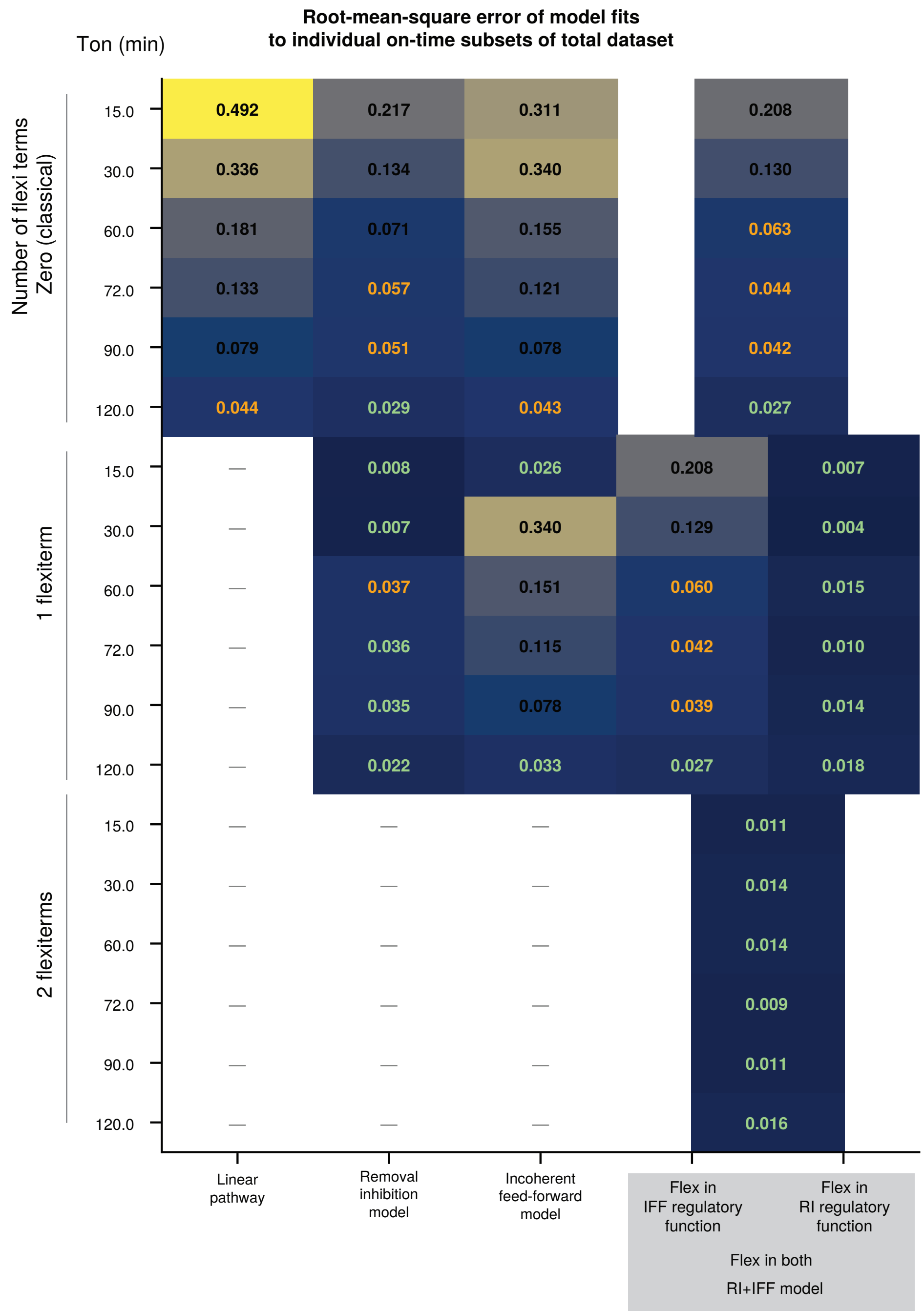

Figure S2: Best-fit rms error of models, fit to individual on-time series, for models with classical mathematical biology functional forms, and 1 or 2 flexifunctions. Text font indicates whether the fit root-mean-square error was smaller than the experimental data's standard deviation (orange) or smaller than the experimental data's standard error of the mean (green). Note that the RI+IFF model, which has both removal-inhibition and incoherent-feedforward regulatory functions, there are two options for where to put a single flexifunction.

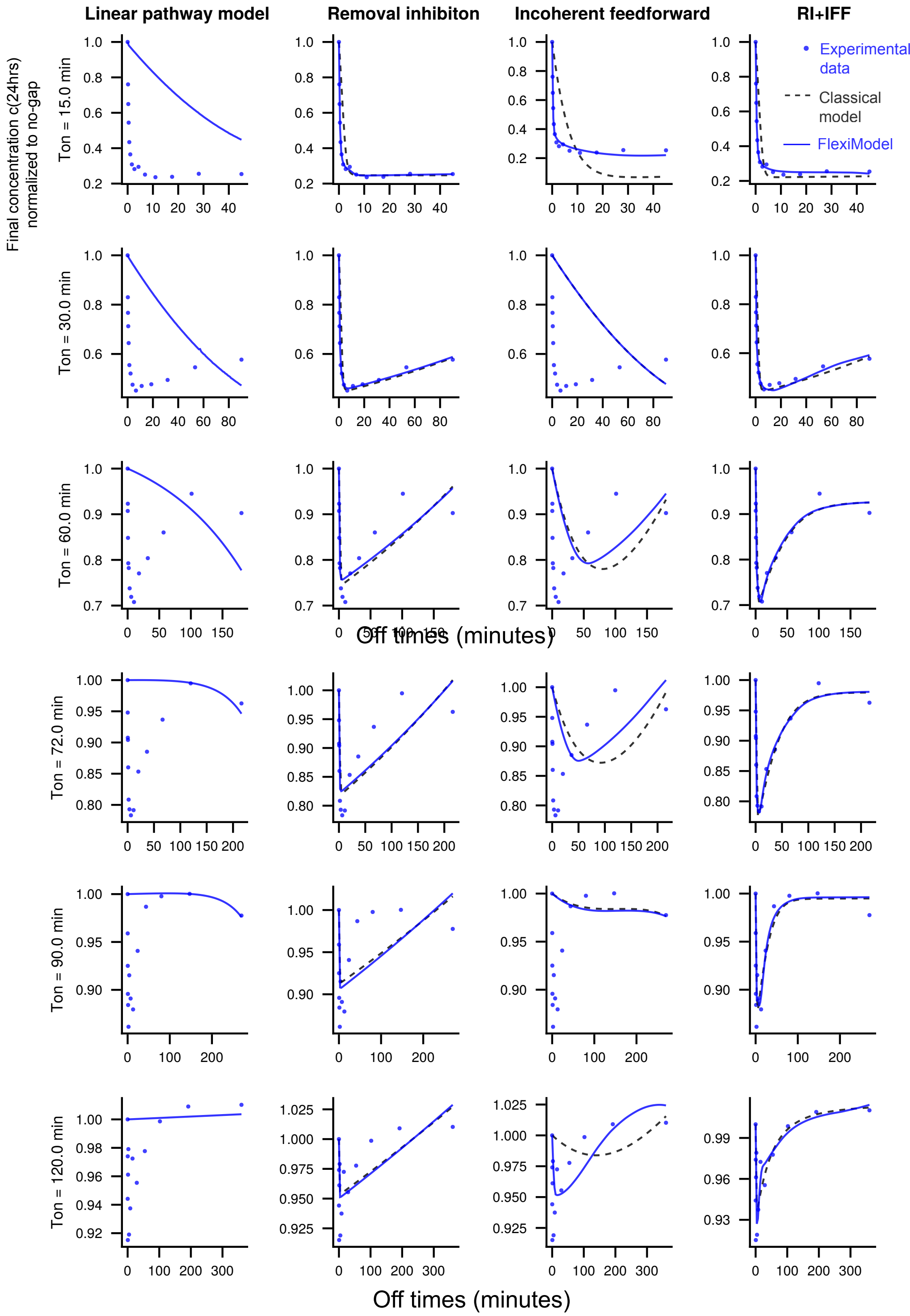

Figure S3: Best-fit rms error of models, fit to individual on-time series, for models with classical mathematical biology functional forms (for 4 models), and 1 flexifunction (for 3 models). These correspond to a subset of rms errors reported in Supplemental Figure S2.

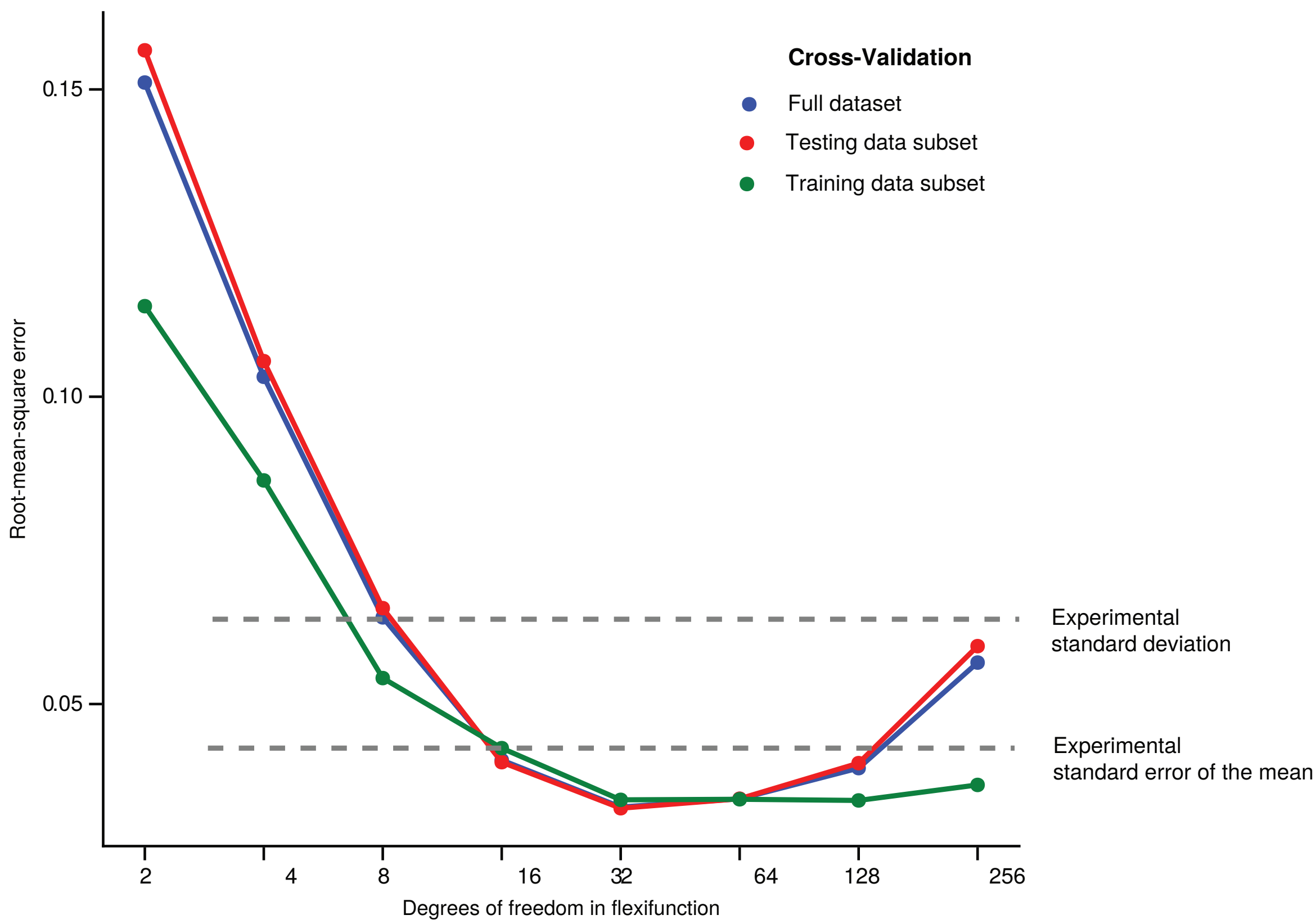

Figure S4: Cross-validation analysis allows choice of degrees of freedom in flexifunctions to avoid over-fitting. RI+IFF model (two flexifunctions) was fit to subsets of the full dataset, specifically 7 subsets were generated where 14 datapoints were removed without replacement (7 was chosen since the total number of datapoints was 7×14). The fit quality, measured by root-mean-square error to the training set (green), generally improved as more degrees of freedom were added to the flexifunctions. Then, the root-mean-square error was measured on the removed subset (testing data subset, red). The error in the testing subset exhibits a pronounced minimum around 32 degrees of freedom. Note importantly that adding more degrees of freedom did not allow unlimited improvements to the fit to the training set — this suggests that these models have structural constraints that would allow a model to be rejected, even with infinite degrees of freedom. The error on the training data is slightly above the testing data for 2 points, which may be due to stochastic optimization. The slight increase in error on the training data at very large degrees of freedom may indicate the algorithm was not yet converged at these very large model sizes.

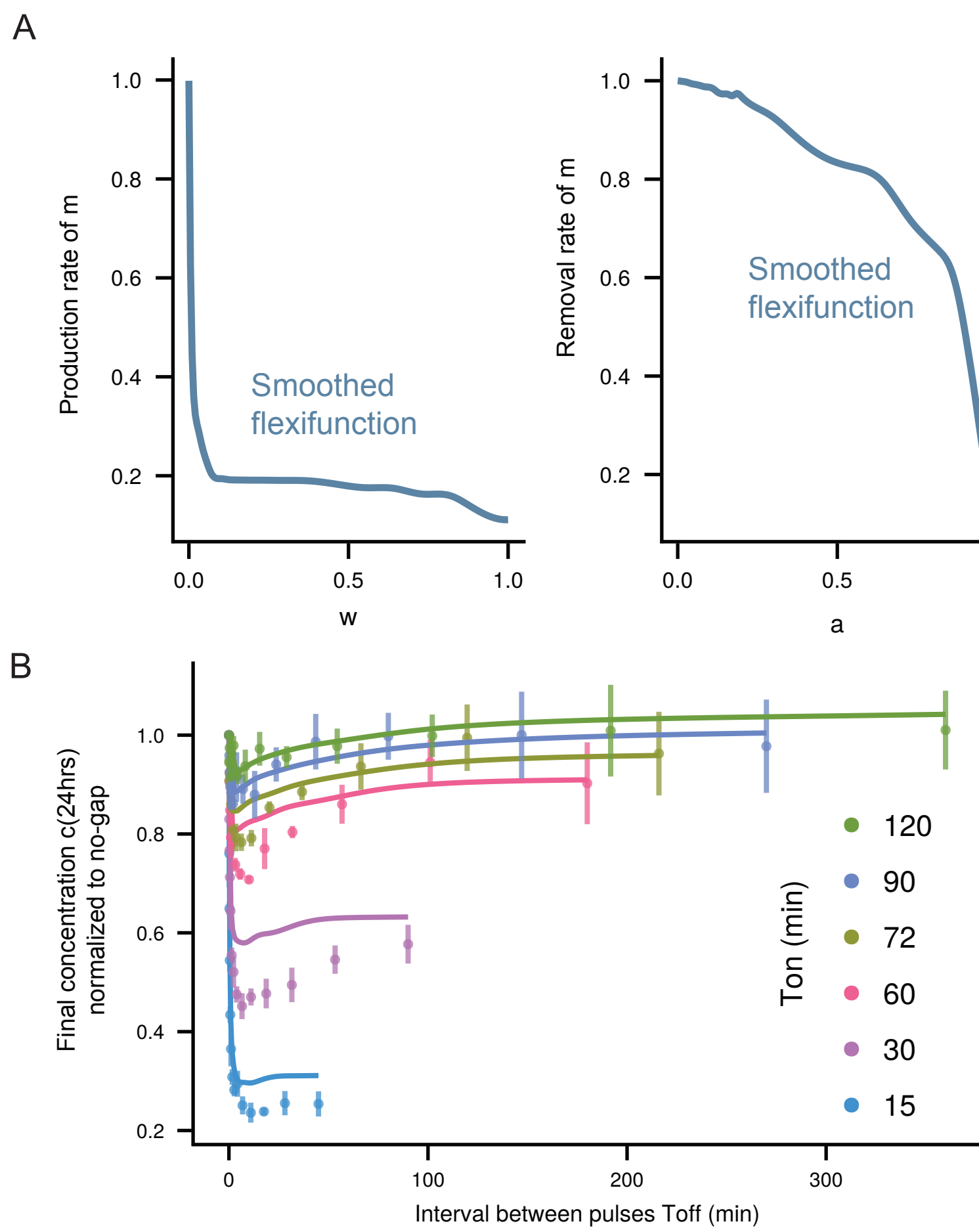

Figure S5: The regulator functions learned from data can be smoothed, leading to minimal impact on qualitative and quantitative consistency with data. A Smoothed regulator functions. Compare with Figure 3B. B The impact of smoothed regulator functions on the predicted frequency response. Note the model was not retrained, so this is a true test of model sensitivity to details of the regulator functions.

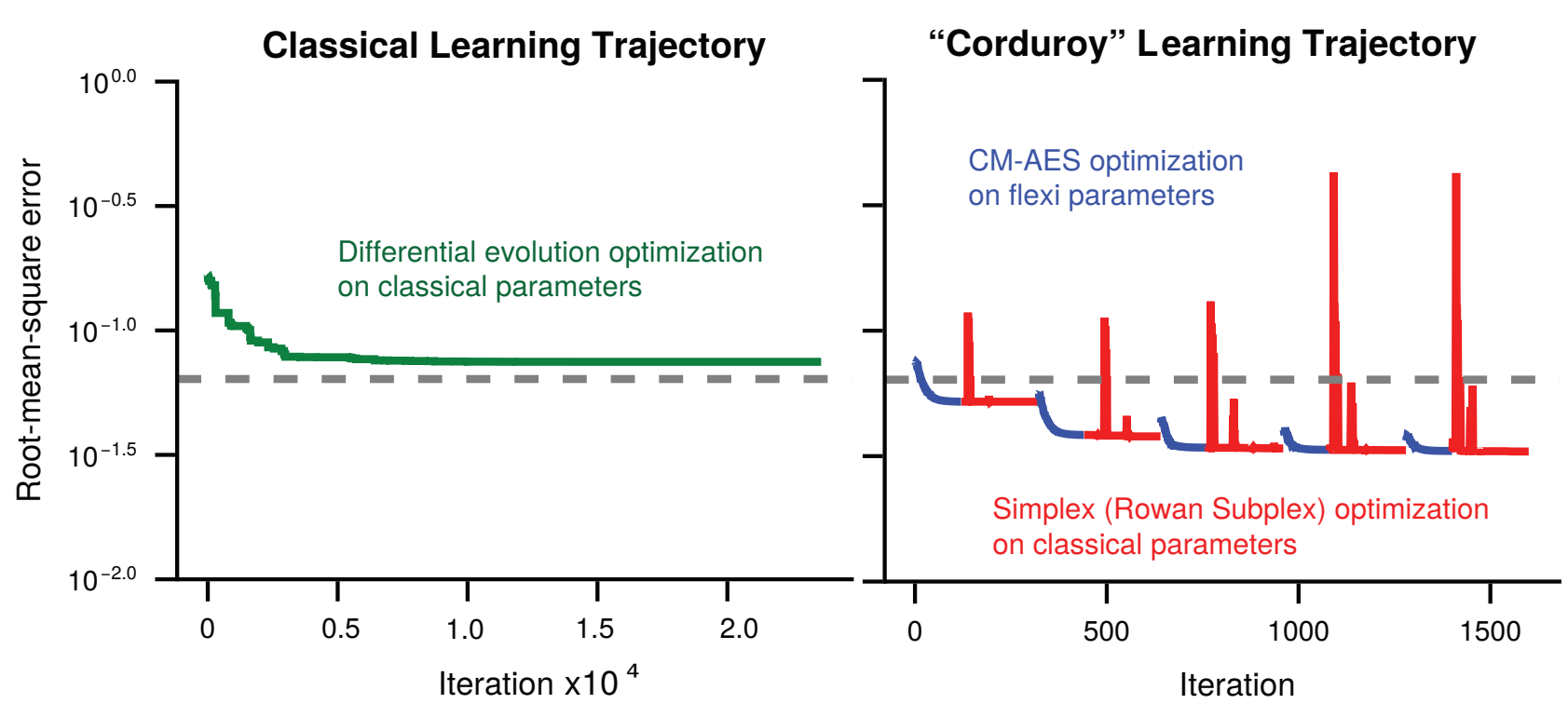

Figure S6: Iterative learning algorithm. To find the optimal parameter set, we first perform differential evolution optimization on just the classical model (left). Then, use these as initial guesses and repeat CMA-ES optimization on only the flexifunction parameters, keeping the classical parameters fixed, and simplex (Rowan's Subplex) on just the classical parameters, keeping the flexifunction parameters fixed (right)

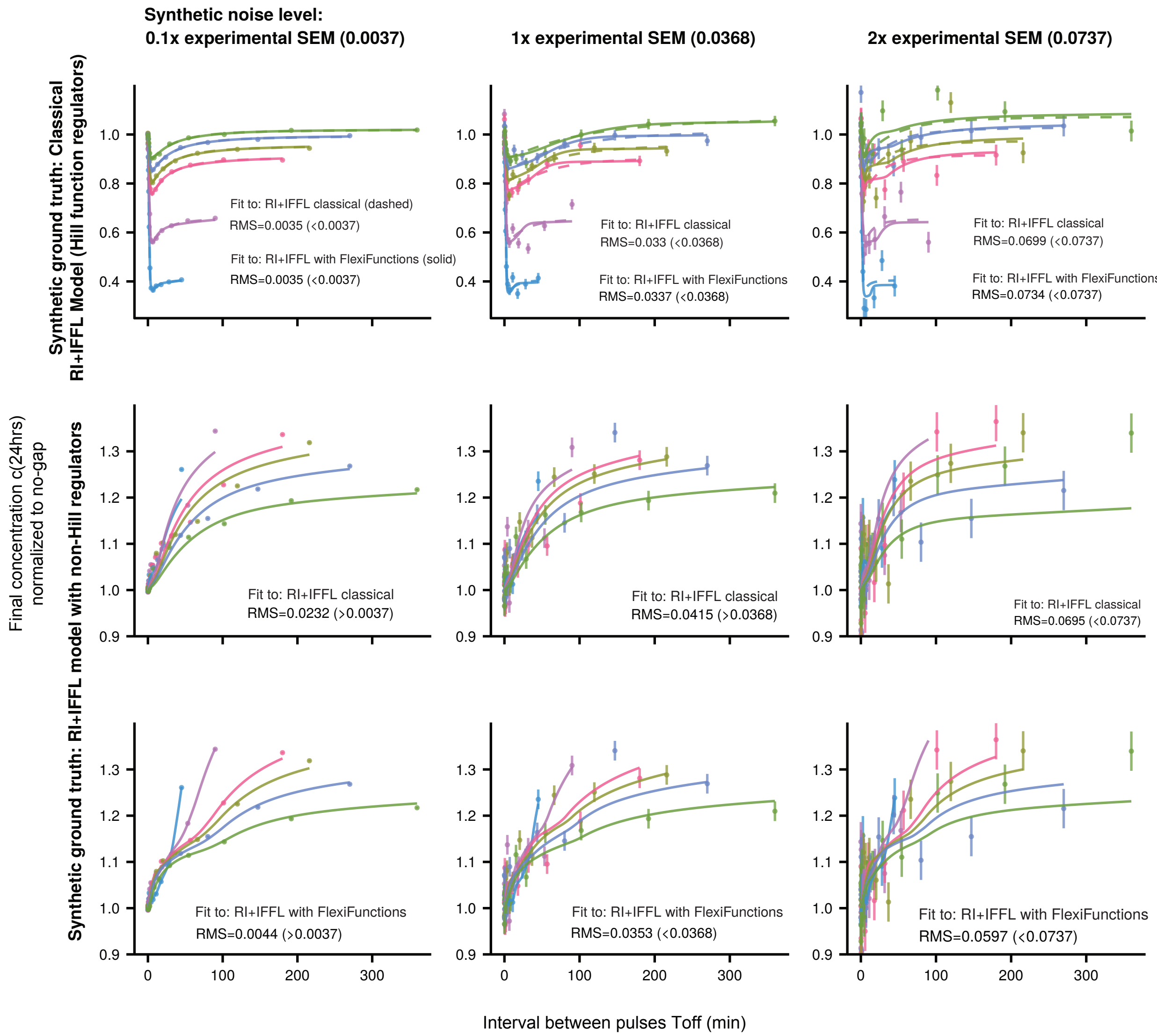

Figure S7: Results of learning on synthetic data in which the ground truth is known. The ground truth is classical RI+IFF model with Hill functions, Equations 15-18 (top row) or RI+IFF model with a non-Hill functions Equation 33-34 for the regulator (middle and bottom row), with three noise levels (columns), corresponding to 10%, 100% and 200% the standard error of the mean experimentally reported in [10]. In each of those 6 synthetic datasets, the classical RI+IFF model with Hill functions is fit (top and middle rows), and the RI+IFF model with flexifunction regulators is fit (top and bottom rows). For classical ground truth, both models fit within the noise. For nonlinear regulatory function model, at the experimental standard error of the mean, the classical model has RMS error larger than the noise, while the fleximodel has RMS error below the noise. The family of curves represent different T_{on}, with the same color legend as Figure 3
